# Iron metabolism and iron supplementation in cancer patients

**DOI:** 10.1007/s00508-015-0842-3

**Published:** 2015-09-15

**Authors:** Heinz Ludwig, Rayko Evstatiev, Gabriela Kornek, Matti Aapro, Thomas Bauernhofer, Veronika Buxhofer-Ausch, Michael Fridrik, Dietmar Geissler, Klaus Geissler, Heinz Gisslinger, Elisabeth Koller, Gerhard Kopetzky, Alois Lang, Holger Rumpold, Michael Steurer, Houman Kamali, Hartmut Link

**Affiliations:** 1 c/o 1. Medizinische Abteilung, Zentrum für Onkologie, Wilhelminen-Krebsforschungsinstitut, Wilhelminenspital, Montleartstraße 37, 1160 Wien, Austria; 2 Universitätsklinik für Innere Medizin III, MedUni, Wien, Austria; 3 Universitätsklinik für Innere Medizin I, MedUni, Wien, Austria; 4 Institut Multidisciplinaire d’Oncologie IMO, Clinique de Genolier, Genolier, Switzerland; 5 Universitätsklinik für Innere Medizin, MedUni, Graz, Austria; 6 Abteilung Interne 1, KH der Elisabethinen, Linz, Austria; 7 Abteilung Interne 3, AKH, Linz, Austria; 8 1. Medizinische Abteilung, Klinikum Klagenfurt, Klagenfurt, Austria; 9 5. Medizinische Abteilung, Krankenhaus Hietzing, Wien, Austria; 10 3. Medizinische Abteilung, Hanusch-Krankenhaus, Wien, Austria; 11 1. Medizinische Abteilung, Landesklinikum St. Pölten, Pölten, Austria; 12 Abteilung für Innere Medizin, Landeskrankenhaus Feldkirch, Feldkirch, Austria; 13 Abteilung für Interne 1, Krankenhaus der Barmherzigen Schwestern, Linz, Austria; 14 Universitätsklinik für Innere Medizin V, MedUni Innsbruck, Innsbruck, Austria; 15 Bristol-Myers Squibb, Wien, Austia; 16 Medizinische Klinik I, Westpfalz-Klinikum, Kaiserslautern, Germany

**Keywords:** Iron deficiency, Functional and absolute iron deficiency, Tumor anemia, Iron supplementation, Recommendations for clinical practice

## Abstract

Iron deficiency and iron deficiency-associated anemia are common complications in cancer patients. Most iron deficient cancer patients present with functional iron deficiency (FID), a status with adequate storage iron, but insufficient iron supply for erythroblasts and other iron dependent tissues. FID is the consequence of the cancer-associated cytokine release, while in absolute iron deficiency iron stores are depleted resulting in similar but often more severe symptoms of insufficient iron supply. Here we present a short review on the epidemiology, pathophysiology, diagnosis, clinical symptoms, and treatment of iron deficiency in cancer patients. Special emphasis is given to intravenous iron supplementation and on the benefits and limitations of different formulations. Based on these considerations and recommendations from current international guidelines we developed recommendations for clinical practice and classified the level of evidence and grade of recommendation according to the principles of evidence-based medicine.

## Introduction

Iron (Fe), an important trace element, plays a vital role in oxygen metabolism, oxygen uptake, and electron transport in mitochondria, energy metabolism, muscle function, and hematopoiesis. Iron, therefore, is essential for physical functioning and wellbeing, but free bivalent iron is quickly oxidized to its trivalent iron, which when available in its free form can induce the formation of free oxygen radicals which may result in tissue damage [1]. Therefore, iron metabolism is tightly controlled to ensure that free iron levels are kept as low as possible, and to guarantee adequate iron absorption, transport, and storage.

In patients with chronic diseases or cancer, iron regulation and homeostasis often are distorted [2]. This may result in insufficient iron supply to erythroblasts with clinical sequelae of iron deficiency such as weakness, fatigue, and impaired physical fitness and wellbeing as well as anemia. Treatment of iron deficiency in non-cancer patients has been shown to reverse these symptoms [3], and emerging data in cancer patients indicate a beneficial effect of this approach [4].

Here, we briefly summarize the pathophysiology of iron metabolism with particular emphasis on cancer and review the available evidence for iron supplementation in patients with functional iron deficiency. Furthermore, we provide suggestions for clinical practice and respond to a series of clinically relevant questions. The issues presented in this report have been discussed during a personal meeting between the authors and thereafter during the preparation of this article after reviewing and assessing the available scientific evidence. Literature research was conducted by searching PubMed Home/Medline, Cochrane Library, and the recommendations of ASCO, NCCN, ESMO, and EORTC between 1990 and March 2015 for relevant key words. Furthermore, relevant abstracts from ASCO, ESMO, EHA, and MASCC meetings between 2003 and March 2015 were used. After preparing a first version of this manuscript, the text was circulated and reviewed and modified by the authors in three rounds. Finally, a consensual wording was agreed for all statements and questions.

## Epidemiology

About 25 % of the world’s population is anemic, half because of iron deficiency. Individuals from developing countries are especially affected by this problem. Data from the USA show a prevalence of less than 1 % of iron deficiency and iron deficiency anemia in men under the age of 50. In men over 50 years of age, iron deficiency is noted in 2–4 % and iron deficiency anemia in 1–2 %. In premenopausal women, the prevalence of these complications is higher because of blood loss during menstruation. Iron deficiency is found in 9–11 % of women between the age of 12 and 49 years and in 5–7 % of women above this age. The corresponding prevalence for iron deficiency anemia is 2–5 % in younger and 2 % in older female populations [5]. For Austria, precise epidemiological data are not available.

The prevalence of anemia in patients with cancer is remarkably high. In the “European Cancer Anaemia Survey”(ECAS) 39 % of cancer patients were anemic at baseline when they were included in the survey. In those receiving chemotherapy incidence of anemia was noted in 67 % of patients at some point during a 6 month surveillance phase (anemia was defined as hemoglobin (Hb) < 12 g/dl) [6]. Similar data have subsequently been obtained in a survey conducted in Austria [7]. Anemia was correlated with low performance status [2], and many patients did not receive anemia therapy.

Presently, only limited data on iron deficiency in cancer patients are available [4, 8–11]. Expectedly, functional iron deficiency (FID) is much more prevalent than absolute iron deficiency. Epidemiological studies on iron deficiency reported prevalence rates for FID varying between 29–46 %, and for iron deficiency-associated anemia between 7–42 % [2, 4, 9–11]. A recent study in patients with different cancer types revealed a very high prevalence of iron deficiency (functional and absolute) in patients with pancreatic cancer (63 %), followed by colorectal (52 %), and lung cancer (51 %) [2]. Most patients with absolute iron deficiency present with anemia [8] and anemia is associated with higher mortality in cancer patients [12], although a causal relationship between anemia and mortality has not been established. Likewise, solid evidence supporting the claim that correction of anemia would improve prognosis is not available.

Several studies confirm a significant correlation between Hb levels and physical fitness and quality of life in cancer patients [6, 13]. In the aforementioned ECAS study, the group of patients with the lowest WHO performance scores also had the lowest Hb levels [6]. In fact, in cancer patients a close correlation between the relevant range of hemoglobin levels (8–14 g/dl) and quality of life was reported.

## Physiology and pathophysiology of iron metabolism

Iron is an essential cofactor for various enzyme systems which can be classified into heme and non-heme proteins. About two third of the body’s total iron content of 4–5 g is bound to heme-proteins, mainly hemoglobin and myoglobin. In both proteins iron is the central atom of heme, a porphyrin structure bound to the protein part of the molecules.

Ferrous (bivalent) iron of red cells circulating through pulmonary capillaries binds oxygen reversibly. Red cells deliver the oxygen to peripheral organs and tissues where oxygen is deployed and carbon dioxide is taken up in return. Hemoglobin serves as an intermediate oxygen storage in muscle cells and provides an oxygen reserve for short-term muscle activity [14]. Other additional iron-dependent functions pertain to oxygen metabolism (mediated by oxydases, peroxydases, catalases, and others) and to mitochondrial electron transfer (cytochromes). Iron-dependent non-heme proteins include enzymes of mitochondrial energy metabolism (such as mitochondrial aconitase or Fe-S complexes as part of the electron transfer chain), and enzymes of DNA synthesis (such as ribonucleotidereductase). In addition, iron-containing proteins are also involved in the synthesis of collagen, tyrosine, and catecholamines [1–15].

Plasma iron levels are regulated by the glycoprotein transferrin which has two high affinity binding sites for trivalent iron. Transferrin, as the main iron transport protein, supplies iron to iron-dependent systems. The uptake of iron into cells is affected by the transferrin receptor (TfR1). By binding to transferrin, iron becomes soluble in plasma, and the synthesis of free oxygen radicals by free trivalent iron is tightly limited. Normally, the iron-saturation of transferrin (TSAT) lies around 30 %; a TSAT below 20 % points to iron deficiency, a TSAT over 45 % to iron overload. Above a TSAT of 60 %, free, non-transferrin-bound iron is released into plasma and damage of parenchymal cells can occur [16].

**Fig. 1:**
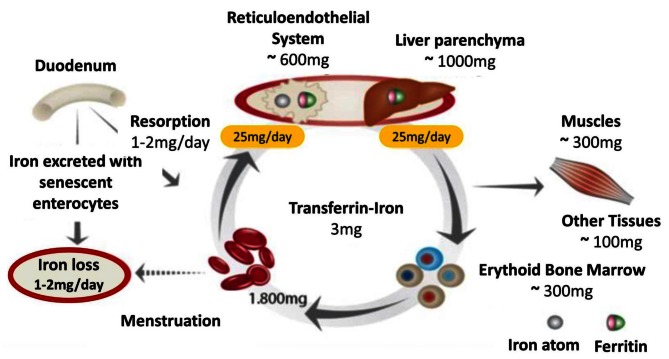
Diagram of iron resorption and metabolism (with kind permission of Evstatiev R, Gasche Ch, and “Gut”)

Iron finds its way into circulation via two paths (Figure 1): duodenal enterocytes absorb 1–2 mg of iron per day from nutrition, and macrophages release 20–25 mg iron per day from internalized senescent red cells. Hepatocytes also play an important role in iron metabolism: They store large amounts of iron via ferritin, and they synthesize and secrete the regulatory protein hepcidin [16]. Hepcidin regulates enteral iron uptake and the release of iron from the reticuloendothelial system (RES) by binding to the only cellular iron exporter, ferroportin, and degrading it [17].

**Fig. 2:**
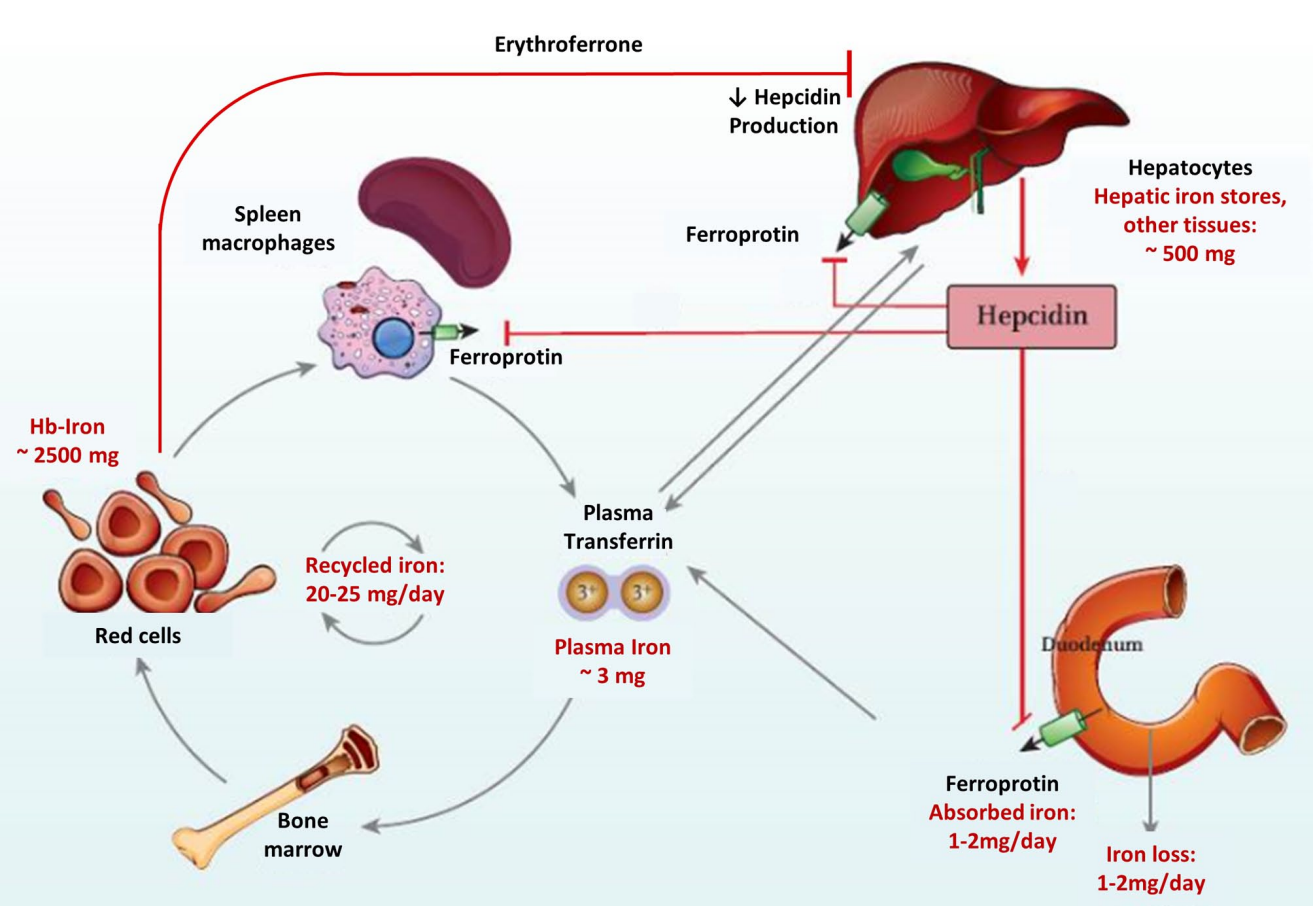
Hepcidin—central regulator of iron uptake and release (with kind permission of Evstatiev R, Gasche Ch, and “Gut”)

Ferroportin is a membrane-bound transport protein, synthesized in liver, spleen, kidneys, heart, gut, and placenta. It is localized in the basolateral membrane of endothelial cells and there affects the efflux of iron into the blood. Ferroportin can only bind and transport ferrous (bivalent) iron. After passing the cell membrane and being oxidized to trivalent Fe by the plasma protein ceruloplasmin or the membrane protein hephestin, Fe can be bound to transferrin and be further transported through the blood (Figure 2).

High hepcidin levels inhibit enteral iron resorption and release of iron from the RES, whereas low hepcidin levels create the opposite effects. Hepcidin production is stimulated by high iron plasma levels and inflammatory processes, whereas low iron plasma levels, hypoxia, and increased erythropoiesis inhibit the expression of hepcidin [17]. The regulation of hepcidin transcription is heavily influenced by the “bone morphogenetic protein” receptor (BMP) which is activated through binding BMP and membrane-bound hemojuvelin (HJV) and then phosphorylates Smad4. Smad4, and RSmad form heterodimers which are translocated into the nucleus where they activate the transcription of the hepcidin-encoding HAMP-gene. Binding of soluble HJV to BMP obviates the formation of membrane-bound BMP-HJV complexes and thus the activation of the BMP receptor. Inflammatory proteins like IL-6 activate Stat3 which can also active the HAMP promotor and thus increase the transcription of hepcidin [17]. Recently, erythroferrone has been described as probably the most important hepcidin regulator. Erythroferrone is produced by erythroblasts in response to erythropoietin. It suppresses hepcidin expression which in turn facilitates release of intracellular iron and enteral iron absorption [18].

## Absolute and functional iron deficiency in cancer patients

Anemia is common in cancer patients [2]. From a practical viewpoint, two forms of iron deficiency need to be differentiated: absolute iron deficiency (AID) and functional iron deficiency (FID). AID is defined by a TSAT < 20 % and a serum ferritin level < 30 ng/ml in otherwise normal individuals, while in cancer patients a higher cut off level of ferritin levels should be applied (< 100 ng/ml). FID is defined by a TSAT < 20 % and serum ferritin levels of > 30 ng/ml in normal individuals and of > 100 ng/ml in cancer patients.

In addition, there are hereditary disorders based on molecular defects of enzymes involved in iron transport, iron recycling, or iron utilization, which all can also lead to iron deficiency but which are not further elaborated on in this context (see Table 1).

**Table 1:** Pathophysiology of iron deficiency. (Source: modified from [17])

**Absolute iron deficiency**	Growth/Development
	In women:
	Pregnancy/breast feeding
	Menstrual blood losses
	Chronic blood loss:
	Blood donation
	Nonsteroidal anti-inflammatory drugs (NSAIDs)
	Gastrointestinal neoplasms, Gastrointestinal parasites (developing countries)
	Angiodysplasia
	Decreased iron absorption:
	Celiac disease
	Helicobacter pylori infection
	Autoimmune atrophic gastritis
	Gastrectomy
**Functional iron deficiency**	Anemia of chronic disease/inflammation:
	Infections
	Malignancies
	Chronic kidney disease
	Autoimmune diseases
	Therapy with erythrocyte-stimulating agents (ESA)
	Hepcidin-producing adenomas
	Iron-refractory iron deficiency anemia (IRIDA)
	Copper deficiency
**Molecular defects in iron transport,recycling, and utilization**	Divalent metal transporter 1 (DMT1) mutations
	Hypotransferrinemia
	Mutations in Ferroportin
	Aceruloplasminemia
	Hereditary sideroblastic anemias (ALAS 2 mutations)
	Heme oxygenase deficiency

Patients with inflammatory bowel diseases often display a combined form of absolute and relative iron deficiency

AID is characterized by both depleted iron stores and insufficient iron supply, while in FID iron stores are loaded, but the deposited iron is unavailable for the erythroblast and for other iron-dependent processes like oxygen transport. This reduction in disposable iron (functional iron deficiency) is mainly mediated by increased hepcidin serum levels, which suppresses the release of adequate quantities of iron into the circulation and which inhibits enteral iron absorption (see Sect. 2) [19, 20].

FID is one of the leading causes for the so-called anemia of chronic disease (ACD). The inadequate iron supply is due the above cited upregulation of hepcidin by pro-inflammatory cytokines such as IL-6, IL-1, TNF-α, and interferon-γ, and anti-inflammatory cytokines such as IL-10 [19, 21].

A reduced erythropoietic activity also might contribute to increased hepcidin levels, by a diminished production of erythroferrone. Hepcidin binds to ferroportin leading to internalization and intracellular degradation of the latter. Hepcidin, is made up of 20 amino acids, and is the master regulator in the development of FID in the context of chronic disease [19] including anemia of cancer, chronic inflammatory bowel disease, rheumatoid arthritis, infections, and other diseases with increased production of inflammatory cytokines [20].

Importantly, several other mechanisms can cause or aggravate anemia in cancer patients. These include acute and/or chronic bleeding, hemolysis, vitamin B12 and folic acid deficiency [20], renal failure, metastatic infiltration of bone marrow, and therapy-related factors like myelosuppression or hemolytic anemia caused by chemo- and/or radiotherapy.

## Diagnosis of iron deficiency

Serum ferritin levels correlate with body iron content in individuals without inflammation or cancer. The amount of biologically available iron is reflected by various parameters such as by transferrin saturation (TSAT), percentage of hypochromic erythrocytes (% HYPO), Hb-content of reticulocytes (CHr), soluble transferrin receptor (sTfR), and sTfR/ferritin index [22]. Ferritin is an acute phase protein, and usually is increased in inflammatory states or in conditions with damaged hepatocytes; this is why in these situations ferritin levels do not correlate with the storage iron, a fact which is especially true for cancer patients [23]. Therefore, presence of inflammation should be assessed by determination of C-reactive protein (CRP) levels. In specific cases cholinesterase, albumin, or INR (International Normalized Ratio) should be determined as well for gross exclusion of relevant liver function impairment.

Further sources of ferritin production are cancer cells which can produce ferritin themselves making them partly independent of external iron supply. This can result in marked increase in serum ferritin levels and in certain cancers ferritin has been shown to closely correlate with tumor stage. Besides its role as indirect tumor marker, ferritin seems to enhance tumor proliferation by stimulating angiogenesis and by suppressing the host’s immune system [24].

The serum levels of the soluble transferrin receptor (sTfR) are strongly increased in patients with AID, but reduced in those with ACD and chemotherapy-induced anemia, making this parameter unsuitable for the assessment of iron homeostasis [25]. As mentioned before, serum ferritin level < 100 ng/ml in combination with a TSAT < 20 % indicate absolute iron deficiency (AID) in cancer patients [23]. The cut off level of serum ferritin for absolute iron deficiency in cancer has been set higher than the one for individuals without cancer (< 30 ng/ml), because of the non-iron specific increase in ferritin levels by subclinical or overt inflammatory processes.

A TSAT < 20 % points to an insufficient availability of iron for iron-dependent processes like erythropoiesis and oxygen transport. In a previous study on the correlation between TSAT and bone marrow iron content in patients with lymphoproliferative diseases, normal iron stores were found in 39 % of patients with low TSAT (< 20 %) indicating the presence of FID in spite of adequate storage iron [4, 11, 26]. These patients were then treated with erythropoietin and randomized into a group that received intravenous iron supplementation and a control group. Patients treated with intravenous iron showed a significant increase in TSAT, whereas TSAT dropped significantly in the control group. A decoupling of ferritin and TSAT was also shown in a different group of patients suffering from either hematological malignancies or solid tumors. In 22 % of patients with a serum ferritin between 100 and 800 ng/ml and in 24 % of patients with a serum ferritin ≥ 800 ng/ml iron deficiency anemia was observed (TSAT < 20 %, Hb < 12 g/dl) [27]. Notably, measurements of TSAT have some limitations. In inflammatory diseases, the reduced expression of transferrin receptors in the diminished pool of erythroblasts can result in a falsely high transferrin saturation [28].

Many modern hematology analyzers provide information about the iron content of red cells and of reticulocytes. More than 5 % of hypochromic red cells and/or less than 26 pg reticulocyte hemoglobin are indicative of iron deficiency. Both tests are cheap and informative, but are rarely used. Serum levels of transferrin receptors (sTfR) are increased in AID, but normal in FID. The ferritin index (sTfR/log ferritin) provides information about body iron stores even in the presence of inflammation when ferritin levels usually are increased because ferritin is a member of the acute phase proteins.

## Clinical consequences of iron deficiency in cancer patients

The most important consequence of Fe deficiency is the risk for developing anemia or the aggravation of already existing anemia. Clinical sequelae of anemia can be manifold with impairment of quality of life being most frequent [13]. This usually is accompanied by deterioration of the performance status [4], and often by inability to adhere to therapy or to the scheduled dose of chemotherapy. These complications usually are associated with impaired outcome.

The mere occurrence of iron deficiency may be associated with several complications. Typical symptoms are pallor, cold skin, weakness and fatigue [29], reduced physical fitness [30], brittle nails, angular cheilosis, impairment of cognitive functions [31], headaches, insomnia, restless legs syndrome, depression, loss of libido, dyspnea, tachycardia, thrombocytosis, increased thromboembolic complications, alopecia, and in rare severe cases Plummer–Vinson syndrome.

## Therapeutic options and goals

The goals of anemia therapy are the avoidance of red blood cell transfusions and the improvement in quality of life. The aims of therapy of iron deficiency are the improvement or avoidance of anemia and the normalization of symptoms due to iron deficiency. For treatment of anemia in cancer patients three principal options are available, namely red blood cell transfusions (RBC), erythropoiesis-stimulating agents (ESA), and iron. The latter two treatments can be combined to enhance the effectiveness of either one.

Blood transfusions should be used diligently because of their possible risk for morbidity and even mortality (increased risk of infection, transfusion reactions, lung injury, alloimmunization, stroke, myocardial infarction, transmission of yet unidentified pathogens, thromboembolic complications, kidney injury, and cancer recurrence) [25, 32–36].

ESA can reduce transfusion requirements in cancer patients [37]. However, only 30–75 % of patients respond to ESA [38–40]. In addition, ESA can lead to an increased rate of thromboembolic events and, if used off-label in patients not currently receiving chemotherapy, to increased mortality [41]. For these reasons, both the European (EMA) and US (FDA) regulatory authorities have strictly limited their use [42–44].

Iron therapy for functional iron deficiency is an established treatment in nephrology but relatively new in cancer. Importantly, oral iron administration is the treatment of choice in asymptomatic or mildly symptomatic patients with FID or AID and no clinical or subclinical inflammation [25, 45]. The latter is a typical phenomenon of cancer, making malignant diseases unsuitable for oral iron therapy. In cancer, enteral iron absorption is severely impaired with more than 95 % being excreted via feces. Furthermore, only an insufficient amount of resorbed iron and intracellular storage iron is available for the erythroblasts because of the cytokine-induced disturbances of iron metabolism as described above. AID should always be treated in cancer and FID if associated with symptoms and/or relevant anemia.

### Available drugs for iron treatment

Table 2 shows a summary of oral and intravenous iron preparations (single agent preparations) available in Austria. Oral preparations can be composed of bivalent or trivalent iron, but in Austria, all available preparations contain bivalent iron, which is better tolerated. Intravenous iron formulations contain trivalent iron bound to carbohydrate or dextran carriers (hydroxide-dextran, hydroxide-saccharose, isomaltoside, and carboxymaltose). High doses of iron (approximately 1 g per infusion) can be administered as single dose with the newer iron preparations ferric carboxymaltose or Fe (III)-or ferric-isomaltoside, thus reducing the need for more hospital visits.

**Table 2:** Iron preparations (monopreparations) licensed in Austria. (Source: Austrian prescribing information (online, as of September 2013); for detailed information on application and contraindications, refer to the respective prescribing information)

*1. Oral preparations*				
	*Galenic*	*Iron content (mg)*	*Registered trade name*	
Iron (Fe)(II)-Fumarate	Capsules	100	Ferretab®	
Fe(II)-Gluconate	Effervescent tablets	100	Loesferron® forte	
Fe(II)-Sulfate	Coated tablets	105	Ferrogradumet®	
Fe(II)-Sulfate	Sustained-release tablets	80	Tardyferon®	
*2. Intravenous preparations*				
*Iron-compound*	*Iron content (mg/ml)*	*Registered trade name*	*Age restriction*	*Maximum single dose*
*2.1 Dextran-based preparations*				
Fe(III)-isomaltoside^a^	100	Monofer®	*Not recommended under 18 years of age*	*1000 mg (20 mg/kg body weight)*
Fe(III)-hydroxide dextrane^c^	50	Cosmofer®	*Not recommended under 14 years of age*	*20 mg/kg body weight*
*2.2 Non-dextran-based preparations*				
Fe(III)-carboxymaltose^c^	50	Ferinject®	*14 years and above*	*1000 mg (20 mg/kg body weight)*
Fe(III)-hydroxide-saccharose^c^	20	Fermed®	*Not recommended in children*	*200 mg*
Fe(III)-hydroxide-saccharose^c^	20	Venofer®		
Fe(III)-hydroxide-saccharose^c^	20	Ferrologic®		

^a^Indication: iron deficiency anemia;

^b^Indication: iron deficiency anemia in chronic kidney disease;

^c^Indication: iron deficiency

Ferric carboxymaltose consists of a ferric hydroxide core encased in a carbohydrate shell and allows controlled iron release to target tissues after intravenous injection. In a single infusion of ferric carboxymaltose approximately 1000 mg of iron can be administered in approximately 15 min without the risk of releasing larger amounts of free, ionized iron into the circulation. Ferric carboxymaltose is rapidly cleared from plasma—approximately 80 % enters the bone marrow and the rest the liver and spleen [46]. Fe (III)-isomaltoside possesses similar favorable properties. In this formulation, iron is bound to a matrix which prevents excessive release of free iron. It can also be administered in doses of up to 1000 mg of iron within a short infusion time.

### Recommendations of current guidelines

Up to now international scientific organizations have not issued specific guidelines for iron supplementation in cancer patients. Only a few comments regarding management of iron deficiency have been made in the context of guidelines for the management of cancer or chemotherapy-induced anemia by the “*National Comprehensive Cancer Network*” *(NCCN)* or in guidelines on the use of ESA by ASH (American Society of Hematology)/ASCO (American Society of Clinical Oncology), EORTC (European Organisation for Research and Treatment of Cancer), and ESMO (European Socierty of Medical Oncology). These are summarized in Table 3 and presented in some detail below.

**Table 3:** Recommendations of international societies on therapy of anemia and iron deficiency in cancer patients and national (Austrian) recommendations for the application of intravenous iron preparations

Society	Recommendations
NCCN [45]	Monotherapy with intravenous Fe in AID (ferritin < 30 ng/ml, TSAT < 20 %) indicated; no studies on iron monotherapy in FID; in patients with ferritin between 30 and 100 ng/ml and TSAT between 20 und 50 %, adequate storage iron can be assumed with the exception of patient receiving ESA which are at risk for FID. In the latter situation iron supplementation is recommended, if the expected benefit is larger than the expected risk. Iron should not be administered to patients with active infection!
ASH/ASCO [41]	Iron monitoring recommended. Iron supplementation is recommended in case of iron deficiency, but satisfactory data for detailed recommendations for iron therapy and monitoring are not available.
ESMO [47]	Periodic monitoring of iron homeostasis; intravenous iron induces greater increases in Hb than oral iron and reduces transfusion need.
EORTC [48]	Administration of iron should be restricted to patients with AID or FID.
Austrian Consensus Group	***AID (TSAT*** < ***20*** % ***and ferritin*** < ***30 ng/ml*** ^a^ ***)*** Oral iron should preferentially be used in non-cancer patients without inflammation (CRP normal), cancer patients in complete remission and patients without inflammatory diseases.
	B) Intravenous iron should be administered in cancer patients with AID ***(TSAT*** < ***20*** % ***and ferritin < 100 µg/L*** ^b^ ***)***
	A) Intravenous iron should be considered in cancer patients symptomatic due to FID
	B) Intravenous iron and ESAs should be considered in patients with chemotherapy-induced anemia and planned or ongoing ESA therapy

*NCCN* National Comprehensive Cancer Network, *AID* absolute iron deficiency, *FID* functional iron deficiency

See above: ^a^ in cancer patients < 100 ng/ml, ^b^ ≥ 100 ng/ml

According to the NCCN guidelines on cancer- and chemotherapy- induced anemia [45], oral or intravenous iron monotherapy should be considered in absolute iron deficiency, defined as ferritin < 30 ng/ml and TSAT < 20 %. In patients with a ferritin level between 30 and 100 ng/ml and a TSAT between 20 and 50 %, iron stores can be considered sufficient if these patients do not receive ESA. If such patients receive ESA, those who respond to that treatment run the risk of developing FID. Such patients likely benefit from treatment with intravenous iron.

In anemic patients with a TSAT < 20 % a combination of intravenous iron and ESA should be used. In patients with a TSAT between 20 and 50 %, the application of intravenous iron on top of ESA should only be considered if the expected benefit clearly outweighs the risks associated with this treatment. Patients with active infections should not receive intravenous iron.

The shared “*American Society of Hematology/American Society of Clinical Oncology clinical practice guideline on the use of epoetin and darbepoetin in adult patients with cancer*” *(update 2010)* [41] demands monitoring of iron homeostasis at the beginning and during the course of ESA therapy; if needed, iron supplementation should be given in order to improve the efficacy of ESAs, reduce the necessary ESA doses and the patient’s symptoms. However, there are insufficient data to recommend optimal timing and the best intervals for iron monitoring.

The guidelines of the “*European Society for Medical Oncology*” *(ESMO)* [47], also from 2010, recommend periodic monitoring of iron homeostasis (iron, CRP, transferrin, and ferritin) as well. Furthermore, they state that intravenous administration of iron in patients with iron deficiency leads to larger Hb increase than oral or no iron supplementation, and that iron supplementation also seems to reduce the number of patients receiving blood transfusions.

Finally, the reappraisal of the ESMO and EORTC guidelines for the management of anemia and iron deficiency in patients with cancer-related or chemotherapy-associated anemia include now clear recommendations for the use of intravenous iron in these patients [48]. Intravenous iron should be considered in patients with Hb ≤ 11 g/dl, or Hb decrease by ≥ 2 g/dl and AID or FID even as sole treatment. If an ESA is considered this should be done before initiation of ESA therapy.

### Current evidence

#### Intravenous iron in combination with ESA

There are currently seven fully published trials comparing the combination of intravenous iron and ESAs with ESAs alone. Six [26, 49–53] show significantly higher hematological response rates, shorter time to response, higher quality of life if measured, reduction in transfusion need, and lower ESA usage with the combined therapy as compared to treatment with ESAs only. In one trial [54] a numerical, but statistically non-significant increase in response rate was noted with intravenous iron/ESA compared to ESA alone [25]. A post hoc analysis of this study showed much lower response rates in patients with high compared to those with low hepcidin levels [55]. Another study showed that the combination of intravenous iron and ESAs might also be more cost effective compared to ESAs alone [56].The increase in response rates by addition of intravenous iron to ESAs depends on the iron status of the included patient cohort. Intravenous iron led to a further increment in response rates of 13–19 % [50–53] when patients without FID were treated; but response rates increased by 34–43 % when patients with FID were included [26, 49]. A meta-analysis of eleven studies including 1681 cancer patients randomizing patients to treatment with an intravenous iron preparation (from different sources) confirmed the activity of intravenous iron either as sole treatment or in combination with ESAs, which were used in 9 out of the 11 trials [57]. With the addition of intravenous iron to ESAs as backbone therapy a significant increase in the hematological response rates was noted in seven trials (relative risk [RR] 1.28). Also a significant reduction of transfusion needs was noted (RR 0.76 with ESA and 0.52 without ESA). The increase in the hematological response rates correlated with the total dose of intravenous iron and was independent of baseline iron status. Mortality and tolerability did not differ between patients with and without intravenous iron.

Another meta-analysis [58] showed similar results: hematological response rates increased by 29 % with the addition of intravenous iron to ESA therapy while treatment with oral iron was not effective. A similar pattern was noted for transfusion requirements. Intravenous iron reduced transfusion need by 23 % but oral iron was ineffective. Use of intravenous iron shortened the time to hematological response by one month without increasing toxicity.

#### Intravenous iron as monotherapy

Studies on the efficacy of intravenous iron as sole treatment for cancer-associated anemia or chemotherapy-induced anemia are scarce. Three small trials showed a significant reduction of transfusion requirements by intravenous administration of iron sucrose in patients with gynecological tumors, receiving chemoradiotherapy or platinum-based chemotherapy (40–64 %, *p* = 0.04 [59], 22.7–63.6 %, *p* < 0.01 [60], and 28.1–56.3 % [61]). In 25 non-iron-deficient anemic patients with a baseline-Hb value of 9.6 g/dl, weekly application of 200 mg iron sucrose through a maximum of 12 weeks led to an increase of Hb concentration by 1.6–2.1 g/dl in patients who had received at least 9 of 12 doses. Five patients were assessed as non-responders to intravenous iron and received blood transfusions [62]. Recently, an observational study with ferric carboxymaltose (FCM) was conducted in 68 hematological–oncological practices in Germany. A total of 639 cancer patients were included, out of which 619 received at least one dose of FCM [63]. Mean Hb levels increased by 1.4 g/dl (*n* = 233) with FCM alone and by 1.6 g/dl (*n* = 46) with FCM plus ESAs. Similar improvements in Hb were seen with FCM in patients with both baseline Hb of ≤ 11 g/dl and serum ferritin ≤ 500 ng/ml and in those with serum ferritin above 500 ng/ml and low transferrin saturation (TSAT < 20 %). Patients with ferritin levels < 500 ng/ml showed a tendency for higher increases in Hb compared to those with higher baseline serum ferritin concentration. FCM was well tolerated. The rate of therapy-related adverse events was 2.3 %. Further studies on the effects of intravenous iron preparations in oncological patients are currently ongoing. Recently, a small prospective randomized trial was published which compared a single dose of intravenous iron (FCM 1000 mg) with no treatment (controls) in anemic patients (Hb 8.5–10.5 g/dl, TSAT < 20 %, Ferritin > 40 ng/ml in men and > 30 ng/ml in women) with low grade non-Hodgkin lymphomas and previous chemotherapy. The rise of the median Hb level was significantly higher in the group with intravenous iron therapy than in the control group (Hb 2.5 vs. 0.9 g/dl, *p* < 0.05) [64].

#### Tolerability and adverse effects of intravenous iron

Iron is an important growth factor for rapidly proliferating cells including bacteria or tumor cells [19, 65]. Hence, it is important to review the safety of intravenous high dose iron supplementation. Currently there is no evidence that intravenous iron may increase the risk for infections or tumor growth in cancer patients, but this issue has not carefully been studied as yet. An in vitro study with breast cancer cell lines reported that intracellular iron depletion by inhibiting transferrin receptor expression with siRNA resulted in increased expression of VEGF and increased angiogenesis thereby creating a favorable environment for tumor proliferation [66]. Intravenous iron supplementation of patients with non-Hodgkin lymphomas had no negative impact on progression-free survival after more than 3 years follow-up [67]. Likewise, no increased risk for cardiovascular complications, tumor incidence, and tumor progression was reported.

In the large observational study with ferric carboxymaltose as sole treatment or in combination with ESAs therapy-related side effects were noted in only 2.3 % of patients. The most frequent complications were nausea and diarrhea. In all, 97 % of the physicians participating in this survey rated the efficacy and tolerability of FCM as “very good” or “good” [63].

In 665 patients with terminal kidney failure and chronic hemodialysis, treated with intravenous iron sucrose, no increased rate of infections was found; on the contrary, this group showed a significantly lower risk for hospitalization caused by infections (*p* < 0.001) and—although not significantly—a lower risk of mortality (*p* = 0.08) compared to the general hemodialysis population [68]. Currently, active infections and intolerance are the main contraindications and limitations of intravenous iron supplementation [3, 25].

## Recommendations for clinical practice

In the following paragraph, the level of evidence and the grade of recommendation is assessed according to an algorithm established by the Oxford Institute of Evidence-Based Medicine [69], which is included in the appendix to this publication.

1. **Diagnosis of iron deficiency and indication for iron supplementation**
***Which parameters of iron metabolism should be measured?***

Ferritin and TSAT should be routinely measured; optionally the following parameters should be determined: percentage of hypochromic red cells (%HYPO), Hb content of reticulocytes (CHr), soluble transferrin receptor (sTfR), and the ferritin index (sTfR/log ferritin).

**Level of Evidence 3, Grade of Recommendation C**

- ***What is the indication for initiation of intravenous iron therapy?***

Intravenous iron therapy should be started in patients with AID independent of the actual Hb level. This is particularly important in cancer patients scheduled for surgery, where iron deficiency should be corrected whenever possible before the planned intervention. FID should be corrected by intravenous iron if patients are symptomatic because of iron deficiency and/or of anemia and in those being scheduled for ESA therapy. In patients with high ferritin levels (> 500 ng/ml) iron supplementation should be based on individual decisions [70]. Intravenous iron supplementation should be withheld in those with ferritin levels > 1000 ng/ml [72].

**Level of Evidence 2b, Grade of Recommendation B**

- ***How does age and comorbidity impact the decision for treating iron deficiency and/or anemia?***

Higher age and coexistent comorbidities increase the risk for symptoms due to AID, FID, and anemia: Iron deficiency should be corrected in all patients symptomatic because of ID. As increasing age and comorbidities do increase the risk for clinical sequelae, treatment should be initiated without delay.

**Level of Evidence 2b, Grade of Recommendation B**

2. **Is there an indication for treating every patient with FID?**

If iron deficiency and anemia do not impair the patient’s quality of life or do not cause any untoward symptoms therapy of FID can be withheld, but the patient should be monitored carefully in order to start therapy if symptoms or anemia occur.

**Level of Evidence 2b, Grade of Recommendation B**

- ***What is the indication for red blood cell transfusions?***

Patients with life threatening anemia or with severe symptoms due to anemia should immediately be treated with RBC transfusions. The trigger Hb level is higher in elderly patients (Hb 8/dl) and between 6–8 g/dl in younger fit patients. Initially, only one unit of RBC should be administered. In case of insufficient improvement a second or rarely a third unit should be given.

In the absence of an urgent need for a rapid increase of Hb levels alternatives to RBC transfusions like ESAs (in chemotherapy-associated anemia) and/or supplementation of iron deficiency (in case of ID) should be considered.

**Level of Evidence 2a, Grade of Recommendation B**

- ***What is the indication for erythropoiesis-stimulating agents (ESAs)?***

Treatment with ESAs should be considered in patients with symptomatic chemotherapy-induced anemia and Hb levels < 10 g/dl. ESA should only be given during ongoing chemotherapy until 4 weeks after discontinuation of chemotherapy. The only exception to this recommendation are patients with low and intermediate risk MDS. Those without cytogenetic abnormalities and endogenous erythropoietin levels < 500 ng/ml are more likely to respond, but response may take longer than in other cancers. The target Hb level is 10–12 g/dl.

**Level of Evidence 1c, Grade of Recommendation A**

- ***What is the indication for intravenous iron as monotherapy?***

Oral iron is a valuable option for non-cancer patients with AID without inflammation, with few symptoms only, and without an urgent need for anemia correction. Similar recommendations apply to cancer patients in complete or very good remission with AID. In patients with active cancer who are symptomatic due to FID, treatment with intravenous iron should be considered.

**Level of Evidence 2b, Grade of Recommendation B**

- ***What is the indication for intravenous iron in combination with ESA?***

In patients who are symptomatic due to anemia and/or iron deficiency fulfilling the criteria for iron deficiency (TSAT < 20 % and ferritin < 500 ng/ml) and scheduled for ESA therapy, concomitant treatment with intravenous iron should be considered.

**Level of Evidence 1a, Grade of Recommendation A**

- ***Which intravenous iron preparation should be used?***

Although newer iron dextran preparations are much safer than the previously used intramuscular iron dextran formulations they tend to show a higher risk profile than the other modern preparations. The newer preparations, like iron carboxymaltose or iron isomaltoside bind iron tightly to their carbohydrate shell resulting in much less release of free iron and potentially toxic free oxygen species. This allows the administration of high doses within one infusion enhancing patient comfort and reducing multiple visits in the clinic.

Recent EMA recommendations do no longer require the application of a test dose of intravenous iron, but require administration of intravenous iron only in institutions, which have adequate emergency procedures in place. Allergic reactions can occur at first administration but also in patients who tolerated the same preparation previously. Care should also be taken that iron will be administered strictly intravenously, since paravenous extravasation of iron preparations may induce severe tissue necrosis.

**Level of Evidence 3, Grade of Recommendation C**

3. **What are the target values of iron therapy?**

TSAT should rise above 20 %, ferritin clearly above 30 ng/ml. Further target parameters are: reduction of the percentage of hypochromic erythrocytes (%HYPO) to < 5 %, increase in Hb of reticulocytes (CHr) to > 28 pg, normalization of levels of soluble transferrin receptor (levels vary depending on the local laboratory), and normalization of the ferritin index (sTfR/log ferritin).

**Level of Evidence 3, Grade of Recommendation C**

4. **What is the optimal duration and dose of intravenous iron therapy?**

Usually a loading dose with 1000 mg of intravenous iron will result in adequate iron supply. If lower doses such as 200 mg per infusion are used, repeated administration in weekly or two-weekly intervals is recommended to increase TSAT > 20 %. Ferritin levels will increase as well, but treatment should be discontinued if ferritin increases above 800 ng/ml. Long-term treatment with intravenous iron should be avoided in patients with cancer as safety data for prolonged use in cancer are not available as yet. There is practical experience with this approach in nephrology where patients frequently receive iron supplementation over several years. So far, in these populations neither an increased infection rate nor an increase in tumor incidence were found [68, 72].

**Level of Evidence 4, Grade of Recommendation C**

5. **Which follow-up examinations are recommended?**

Pharmacoepidemiological studies indicate that iron status in cancer patients frequently is not assessed [71]. Parameters that should be evaluated are those of iron metabolism as mentioned above, hemoglobin and red cell parameters. The outcome of intravenous iron supplementation should be controlled only after 2–4 weeks of iron administration (Figs. 1, 2).

### Conflict of interest

The authors declare that there are no actual or potential conflicts of interest in relation to this article.

## References

[1] Ponka P (1999). Cellular iron metabolism. Kidney Int.

[2] Ludwig H, Muldur E, Endler G (2013). Prevalence of iron deficiency across different tumors and its association with poor performance status, disease status and anemia. Ann Oncol.

[3] Anker SD, Comin Colet J, Filippatos G (2009). Ferric carboxymaltose in patients with heart failure and iron deficiency. N Engl J Med.

[4] Ludwig H, Müldür E, Endler G, et al. High prevalence of iron deficiency across different tumors correlates with anemia, increases during cancer treatment and is associated with poor performance status. Haematologica. 2011;96(Suppl 2):409.

[5] Looker AC, Dallman PR, Carroll MD (1997). Prevalence of iron deficiency in the United States. JAMA.

[6] Ludwig H, Van Belle S, Barrett-Lee P (2004). The European Cancer Anaemia Survey (ECAS): a large, multinational, prospective survey defining the prevalence, incidence, and treatment of anaemia in cancer patients. Eur J Cancer.

[7] Steurer M, Wagner H, Gastl G (2004). Prevalence and management of anaemia in haematologic cancer patients receiving cyclic nonplatinum chemotherapy: results of a prospective national chart survey. Wien Klin Wochenschr.

[8] Beale AL, Penney MD, Allison MC (2005). The prevalence of iron deficiency among patients presenting with colorectal cancer. Colorectal Dis.

[9] Kuvibidila SR, Gauthier T, Rayford W (2004). Serum ferritin levels and transferrin saturation in men with prostate cancer. J Natl Med Assoc.

[10] Steinmetz HT, Tsamaloukas A, Schmitz S (2010). A new concept for the differential diagnosis and therapy of anaemia in cancer patients. Support Care Cancer.

[11] Beguin Y, Lybaert W, Bosly A. A prospective observational study exploring the impact of iron status on response to darbepoetin alfa in patients with chemotherapy induced anemia. Blood. 2009;114:2007.

[12] Caro JJ, Salas M, Ward A (2001). Anemia as an independent prognostic factor for survival in patients with cancer: a systemic, quantitative review. Cancer.

[13] Crawford J, Cella D, Cleeland CS (2002). Relationship between changes in hemoglobin level and quality of life during chemotherapy in anemic cancer patients receiving epoetin alfa therapy. Cancer.

[14] Hahn A (2009). Physiologische Bedeutung von Eisen. Essenziell und toxisch. Pharm Unserer Zeit.

[15] Evstatiev R, Gasche C (2012). Iron sensing and signalling. Gut.

[16] Hentze MW, Muckenthaler MU, Galy B (2010). Two to tango: regulation of mammalian iron metabolism. Cell.

[17] Goodnough LT, Nemeth E, Ganz T (2010). Detection, evaluation, and management of iron-restricted erythropoiesis. Blood.

[18] Kautz L, Jung G, Valore EV (2014). Identification of erythroferrone as an erythroid regulator of iron metabolism. Nat Genet.

[19] Weiss G, Goodnough LT (2005). Anemia of chronic disease. N Engl J Med.

[20] Grotto HZ (2008). Anaemia of cancer: an overview of mechanisms involved in its pathogenesis. Med Oncol.

[21] Tilg H, Ulmer H, Kaser A (2002). Role of IL-10 for induction of anemia during inflammation. J Immunol.

[22] Wish JB (2006). Assessing iron status: beyond serum ferritin and transferrin saturation. Clin J Am Soc Nephrol.

[23] Beguin Y (2002). Prediction of response and other improvements on the limitations of recombinant human erythropoietin therapy in anemic cancer patients. Haematologica.

[24] Alkhateeb AA, Connor JR (2013). The significance of ferritin in cancer: anti-oxidation, inflammation and tumorigenesis. Biochim Biophys Acta.

[25] Aapro M, Osterborg A, Gascon P (2012). Prevalence and management of cancer-related anaemia, iron deficiency and the specific role of i.v. iron. Ann Oncol.

[26] Hedenus M, Birgegard G, Nasman P (2007). Addition of intravenous iron to epoetin beta increases hemoglobin response and decreases epoetin dose requirement in anemic patients with lymphoproliferative malignancies: a randomized multicenter study. Leukemia.

[27] Ludwig H, Endler G, Hübl W, et al. High prevalence of iron deficiency in patients with various hematological and malignant diseases: a single center study in 1989 sequential patients. Haematologica. 2010;95(Suppl 2):702.

[28] Stein J, Hartmann F, Dignass AU (2010). Diagnosis and management of iron deficiency anemia in patients with IBD. Nat Rev Gastroenterol Hepatol.

[29] Verdon F, Burnand B, Stubi CL (2003). Iron supplementation for unexplained fatigue in non-anaemic women: double blind randomised placebo controlled trial. BMJ.

[30] Brownlie Tt, Utermohlen V, Hinton PS (2004). Tissue iron deficiency without anemia impairs adaptation in endurance capacity after aerobic training in previously untrained women. Am J Clin Nutr.

[31] Bruner AB, Joffe A, Duggan AK (1996). Randomised study of cognitive effects of iron supplementation in non-anaemic iron-deficient adolescent girls. Lancet.

[32] Vamvakas EC, Blajchman MA (2009). Transfusion-related mortality: the ongoing risks of allogeneic blood transfusion and the available strategies for their prevention. Blood.

[33] Marik PE, Corwin HL (2008). Efficacy of red blood cell transfusion in the critically ill: a systematic review of the literature. Crit Care Med.

[34] Thomson A, Farmer S, Hofmann A (2009). Patient blood managementa new paradigm for transfusion medicine?. ISBT Science Series.

[35] Rawn J (2008). The silent risks of blood transfusion. Curr Opin Anaesthesiol.

[36] Amato AC, Pescatori M (1998). Effect of perioperative blood transfusions on recurrence of colorectal cancer: meta-analysis stratified on risk factors. Dis Colon Rectum.

[37] Bohlius J, Schmidlin K, Brillant C, et al. Erythropoietin or darbepoetin for patients with cancer–meta-analysis based on individual patient data. Cochrane Database Syst Rev. 2009;(3):CD007303. 10.1002/14651858.CD007303.pub2 PMC7208183 19588423

[38] Gabrilove JL, Cleeland CS, Livingston RB (2001). Clinical evaluation of once-weekly dosing of epoetin alfa in chemotherapy patients: improvements in hemoglobin and quality of life are similar to three-times-weekly dosing. J Clin Oncol.

[39] Littlewood TJ, Bajetta E, Nortier JW (2001). Effects of epoetin alfa on hematologic parameters and quality of life in cancer patients receiving nonplatinum chemotherapy: results of a randomized, double-blind, placebo-controlled trial. J Clin Oncol.

[40] Ludwig H, Aapro M, Bokemeyer C (2009). Treatment patterns and outcomes in the management of anaemia in cancer patients in Europe: findings from the anaemia cancer treatment (ACT) study. Eur J Cancer.

[41] Rizzo JD, Brouwers M, Hurley P (2010). American society of hematology/American society of clinical oncology clinical practice guideline update on the use of epoetin and darbepoetin in adult patients with cancer. Blood.

[42] EMA. Summary of Scientific Discussion. http://www.ema.europa.eu/docs/en_GB/document_library/EPAR_-_Scientific_Discussion_-_Variation/human/000332/WC500026146.pdf. Accessed 2015.

[43] FDA. Procrit Label, Epogen Label. http://www.accessdata.fda.gov/drugsatfda_docs/label/2010/103234s5199lbl.pdf. Accessed 2015.

[44] FDA. Aranesp (darbepoetin alfa) for injection. http://www.accessdata.fda.gov/drugsatfda_docs/label/2010/103951s5197lbl.pdf. Accessed 2015.

[45] NCCN. Cancer- and chemotherapy-induced anemia, Version 2.2014. http://www.nccn.org/professionals/physician_gls/pdf/anemia.pdf (2014). Accessed 2015 .

[46] Lyseng-Williamson KA, Keating GM (2009). Ferric carboxymaltose: a review of its use in iron-deficiency anaemia. Drugs.

[47] Schrijvers D, De Samblanx H, Roila F (2010). Erythropoiesis-stimulating agents in the treatment of anaemia in cancer patients: ESMO Clinical Practice Guidelines for use. Ann Oncol.

[48] Aapro M, Beguin Y, Bokemeyer C, et al. A reappraisal of the ESMO and EORTC guidelines for the management of anaemia and iron deficiency in patients with cancer-related or chemotherapy-induced anaemia. Ann Oncol. 2015.

[49] Auerbach M, Ballard H, Trout JR (2004). Intravenous iron optimizes the response to recombinant human erythropoietin in cancer patients with chemotherapy-related anemia: a multicenter, open-label, randomized trial. J Clin Oncol.

[50] Henry DH, Dahl NV, Auerbach M (2007). Intravenous ferric gluconate significantly improves response to epoetin alfa versus oral iron or no iron in anemic patients with cancer receiving chemotherapy. Oncologist.

[51] Bastit L, Vandebroek A, Altintas S (2008). Randomized, multicenter, controlled trial comparing the efficacy and safety of darbepoetin alpha administered every 3 weeks with or without intravenous iron in patients with chemotherapy-induced anemia. J Clin Oncol.

[52] Pedrazzoli P, Farris A, Del Prete S (2008). Randomized trial of intravenous iron supplementation in patients with chemotherapy-related anemia without iron deficiency treated with darbepoetin alpha. J Clin Oncol.

[53] Auerbach M, Silberstein PT, Webb RT (2010). Darbepoetin alfa 300 or 500 mug once every 3 weeks with or without intravenous iron in patients with chemotherapy-induced anemia. Am J Hematol.

[54] Steensma DP, Sloan JA, Dakhil SR (2011). Phase III, randomized study of the effects of parenteral iron, oral iron, or no iron supplementation on the erythropoietic response to darbepoetin alfa for patients with chemotherapy-associated anemia. J Clin Oncol.

[55] Steensma DP, Sasu BJ, Sloan JA (2011). The relationship between serum hepcidin levels and clinical outcomes in patients with chemotherapy-associated anemia treated in a controlled trial. ASCO Meeting Abstracts.

[56] Luporsi E, Mahi L, Morre C (2012). Evaluation of cost savings with ferric carboxymaltose in anemia treatment through its impact on erythropoiesis-stimulating agents and blood transfusion: french healthcare payer perspective. J Med Econ.

[57] Gafter-Gvili A, Rozen-Zvi B, Vidal L (2013). Intravenous iron supplementation for the treatment of chemotherapy-induced anaemiasystematic review and meta-analysis of randomised controlled trials. Acta Oncol.

[58] Petrelli F, Borgonovo K, Cabiddu M (2012). Addition of iron to erythropoiesis-stimulating agents in cancer patients: a meta-analysis of randomized trials. J Cancer Res Clin Oncol.

[59] Kim YT, Kim SW, Yoon BS (2007). Effect of intravenously administered iron sucrose on the prevention of anemia in the cervical cancer patients treated with concurrent chemoradiotherapy. Gynecol Oncol.

[60] Dangsuwan P, Manchana T (2010). Blood transfusion reduction with intravenous iron in gynecologic cancer patients receiving chemotherapy. Gynecol Oncol.

[61] Athibovonsuk P, Manchana T, Sirisabya N (2013). Prevention of blood transfusion with intravenous iron in gynecologic cancer patients receiving platinum-based chemotherapy. Gynecol Oncol.

[62] Abdel-Razeq H, Abbasi S, Saadi I (2013). Intravenous iron monotherapy for the treatment of non-iron-deficiency anemia in cancer patients undergoing chemotherapy: a pilot study. Drug Des Devel Ther.

[63] Steinmetz T, Tschechne B, Harlin O (2013). Clinical experience with ferric carboxymaltose in the treatment of cancer- and chemotherapy-associated anaemia. Ann Oncol.

[64] Hedenus M, Karlsson T, Ludwig H, et al. Intravenous ferric carboxymaltose as sole anemia therapy in patients with lymphoid malignancies, chemotherapy-induced anemia and functional iron deficiency Blood. 2013;122:3439.

[65] Gilreath JA, Stenehjem DD, Rodgers GM (2012). Total dose iron dextran infusion in cancer patients: is it SaFe2 +. ?. J Natl Compr Canc Netw.

[66] Eckard J, Dai J, Wu J (2010). Effects of cellular iron deficiency on the formation of vascular endothelial growth factor and angiogenesis. Iron deficiency and angiogenesis. Cancer Cell Int.

[67] Beguin Y, Maertens J, De Prijck B, et al. Darbepoetin-alfa and I.V. iron administration after autologous hematopoietic stem cell transplantation: a prospective randomized multicenter trial. Am J Hematol. 2013;88(12):990-6. 10.1002/ajh.23552 23873823

[68] Aronoff GR, Bennett WM, Blumenthal S (2004). Iron sucrose in hemodialysis patients: safety of replacement and maintenance regimens. Kidney Int.

[69] Centre for Evidence Based Medicine (CEBM). Levels of Evidence. http://www.cebm.net/index.aspx?o=1025 (2009). Accessed 2015.

[70] KDOQI (2006). Clinical practice guidelines and clinical practice recommendations for anemia in chronic kidney diseaseCPG and CPR 3.2. Using iron agents. Am J Kidney Dis.

[71] Aapro MS, Beguin Y, Bokemeyer C (2011). Diagnosis, treatment, and use of intravenous iron for chemotherapy-induced anemia in Europe. J Clin Oncol.

[72] Kalantar-Zadeh K, McAllister CJ, Regidor DL (2005). Time-dependent associations between iron and mortality in hemodialysis patients. J Am Soc Nephrol.

